# Nuclear PTEN Regulates Thymidylate Biosynthesis in Human Prostate Cancer Cell Lines

**DOI:** 10.3390/metabo13080939

**Published:** 2023-08-11

**Authors:** Zoe N. Loh, Mu-En Wang, Changxin Wan, John M. Asara, Zhicheng Ji, Ming Chen

**Affiliations:** 1 Department of Pathology, Duke University School of Medicine, Durham, NC 27710, USA; 2 Duke Cancer Institute, Duke University, Durham, NC 27710, USA; 3 Department of Biostatistics and Bioinformatics, Duke University School of Medicine, Durham, NC 27710, USA; 4 Division of Signal Transduction, Beth Israel Deaconess Medical Center and Department of Medicine, Harvard Medical School, Boston, MA 02215, USA

**Keywords:** nuclear PTEN, metabolic compartmentalization, thymidylate biosynthesis, cancer

## Abstract

The phosphatase and tensin homologue deleted on chromosome 10 (PTEN) tumor suppressor governs a variety of biological processes, including metabolism, by acting on distinct molecular targets in different subcellular compartments. In the cytosol, inactive PTEN can be recruited to the plasma membrane where it dimerizes and functions as a lipid phosphatase to regulate metabolic processes mediated by the phosphatidylinositol 3-kinase (PI3K)/AKT/mammalian target of rapamycin complex 1 (mTORC1) pathway. However, the metabolic regulation of PTEN in the nucleus remains undefined. Here, using a gain-of-function approach to targeting PTEN to the plasma membrane and nucleus, we show that nuclear PTEN contributes to pyrimidine metabolism, in particular *de novo* thymidylate (dTMP) biosynthesis. PTEN appears to regulate dTMP biosynthesis through interaction with methylenetetrahydrofolate dehydrogenase 1 (MTHFD1), a key enzyme that generates 5,10-methylenetetrahydrofolate, a cofactor required for thymidylate synthase (TYMS) to catalyze deoxyuridylate (dUMP) into dTMP. Our findings reveal a nuclear function for PTEN in controlling dTMP biosynthesis and may also have implications for targeting nuclear-excluded PTEN prostate cancer cells with antifolate drugs.

## 1. Introduction

*Phosphatase and tensin homologue deleted on chromosome 10 (PTEN)* is among the most frequently lost or mutated tumor-suppressor genes in human cancer [1]. PTEN maintains cellular, tissue, and organismal homeostasis by interacting with various molecular targets involved in cell metabolism, growth, survival, and migration in different subcellular compartments [1]. A cornerstone of PTEN biology is its function as a lipid phosphatase for phosphatidylinositol (PtdIns)-3,4,5-trisphosphate (PIP_3_) in the cytoplasm, by which it opposes the activation of the proto-oncogenic phosphatidylinositol 3-kinase (PI3K)/AKT/mammalian target of the rapamycin complex 1 (mTORC1) signaling pathway [2,3,4,5]. PTEN also exerts part of its tumor-suppressive function in the nucleus, mainly by acting as a scaffold protein independently of its lipid phosphatase activity [1]. The absence of nuclear PTEN is associated with more aggressive cancers [6,7,8], suggesting that its function in the nucleus is as relevant to tumor suppression as modulation of the PI3K/AKT signaling in the plasma membrane. Nuclear PTEN regulates diverse processes, including maintenance of genomic stability, transcription, and cell cycle entry [9,10,11,12]. Accordingly, *PTEN* loss does not always correlate with AKT overexpression, as revealed by in vivo genetic analyses of genetically engineered mouse models [13].

Metabolic reprogramming, whether induced by the loss of tumor-suppressor genes or aberrant activation of oncogenes, contributes to tumorigenesis and holds significant promise for targeted cancer therapy [14]. Through antagonizing the PI3K/AKT/mTORC1 pathway in the cytoplasm, PTEN influences a plethora of metabolic processes involved in cell growth and proliferation [15]. The best-characterized impact of PTEN on cell metabolism is its modulation of glucose metabolism. Heterozygous *Pten* inactivation enhances glucose uptake and promotes insulin hypersensitivity in mice, presumably due to increased membrane translocation of the glucose transporter GLUT4 through inhibition of the RAB-GTPase-activating protein AS160, which is an AKT substrate [16,17,18]. Additionally, *PTEN* loss and AKT activation promote ATP hydrolysis, leading to a compensatory increase in aerobic glycolysis (also known as the Warburg effect), presumably due to upregulation of ectonucleoside triphosphate diphosphohydrolase 5 in the endoplasmic reticulum [19]. By contrast, the systemic elevation of *Pten* in mice induces a tumor-suppressive “anti-Warburg” state, characterized by decreased glycolysis but increased oxidative phosphorylation [20,21]. Through mTORC1-dependent upregulation of lipogenic enzymes and S-adenosylmethionine decarboxylase 1, *PTEN* loss is also reported to be involved in lipid and polyamine biosynthesis [22,23]. 

Recently, the nucleus has been proposed as a central hub for metabolism that allows for local control of the core metabolites needed for cell growth/division and the regulation of the epigenome [24]. Many metabolic enzymes translocate to the nucleus to carry out their non-metabolic and metabolic functions [24]. Additionally, certain metabolic activities occur predominantly in the nucleus. For example, thymidylate (dTMP) is synthesized *de novo* within the nucleus by thymidylate synthase (TYMS) from deoxyuridylate (dUMP) with 5,10-methylenetetrahydrofolate as the one-carbon donor [25]. Impaired dTMP biosynthesis results in increased rates of uracil misincorporation into the genome, leading to genomic instability and “thymineless cell death” [26,27,28], an attractive therapeutic target that has been harnessed by antifolate treatment against various forms of cancer [25]. However, whether PTEN regulates metabolism in the nucleus, and what metabolic vulnerabilities may exist in cells lacking nuclear PTEN, remain unknown.

In this study, we demonstrate that one of the top metabolic pathways regulated by nuclear PTEN is pyrimidine metabolism, in particular *de novo* dTMP biosynthesis. PTEN appears to regulate dTMP biosynthesis through interaction with methylenetetrahydrofolate dehydrogenase 1 (MTHFD1), a key enzyme in dTMP biosynthesis. Given that *de novo* dTMP biosynthesis is tightly linked with antifolate treatment, our results may have implications for targeting nuclear-excluded PTEN prostate cancer cells with antifolate drugs.

## 2. Materials and Methods

### 2.1. Plasmids, Reagents, and Antibodies

Human wild type (Wt), N-terminal myristoylated (myr)-tagged, and C-terminal NLS-tagged PTEN complementary DNA (cDNA) sequences were subcloned from pCDNA3-PTEN into pTRIPZ vector (Addgene, Watertown, MA, USA) with AgeI and MluI to generate PTEN expression plasmid. All plasmid constructs were confirmed by sequencing. Ampicillin, puromycin, and doxycycline were purchased from Sigma-Aldrich (St. Louis, MO, USA). Polyethylenimine (PEI) was purchased from Polysciences (Warrington, PA, USA), while RPMI, DMEM, Opti-MEM reduced serum media, and fetal bovine serum (FBS) were purchased from Thermo Fisher Scientific (Waltham, MA, USA), and Protein G Sepharose 4 Fast Flow beads were purchased from Cytiva (Malborough, MA, USA). The antibodies for Western blotting were as follows: anti-PTEN (138G6, 1:1000), anti-p-AKT (9271, 1:1000), anti-AKT (9272, 1:1000), anti-GAPDH (D16H11, 1:4000), anti-Lamin B1 (D4Q4Z, 1:1000), anti-β-Tubulin (9F3, 1:1000), and anti-EGFR (D3871, 1:1000) antibodies, all purchased from Cell Signaling Technology (Danvers, MA, USA). Anti-HSP90 (610418, 1:4000) was purchased from BD Bioscience (Franklin Lakes, NJ, USA). 

### 2.2. Cell Culture and Transfection, Lentiviral Transduction, and Stable Cell Line Establishment

PC3, C4-2, and 293T cells were purchased from ATCC. Cells were checked for mycoplasma using the MycoAlert Mycoplasma Detection Kit (Lonza, Morristown, NJ, USA). Prostate cancer cells were cultured in RPMI medium. 293T cells were cultured in Dulbecco’s Modified Eagle Medium (DMEM). Complete growth media were supplied with 10% fetal bovine serum, 2 mM glutamine, and 100 U/mL of penicillin-streptomycin (Thermo Fisher Scientific). All cells were maintained at 37 °C with 5% CO_2_. To establish inducible PTEN overexpressed cells, 293T cells were used for virus packing due to their expression of SV40 T-antigen, allowing more efficient retroviral production. Briefly, pTRIPZ empty vector or pTRIPZ-PTEN constructs, psPAX2, pMD2.G plasmids, and PEI (DNA:PEI = 1:4) were mixed in Opti-MEM (Thermo Fisher Scientific) for 15 min and transfected into 293T cells. At 48 and 72 h after transfection, the virus-containing medium was collected and filtered with a 0.45 μm filter. The virus-containing suspension was mixed with fresh culture medium at 1:1 ratio and supplemented with 4 μg/mL of polybrene (Santa Cruz Biotechnology, Dallas, TX, USA), and then applied to the cells. A total of 48 h after virus infection, cells were selected using puromycin for 48 h. Cells were pre-treated with 100 ng/mL of doxycycline for 48 h prior to downstream assays. 

### 2.3. Western Blotting, Cell Fractionation, and Immunoprecipitation

For regular Western blotting, cells were lysed in RIPA buffer (Boston BioProducts, Milford, MA, USA) supplemented with protease (Roche, Indianapolis, IN, USA) and phosphatase (Sigma-Aldrich) inhibitor. The protein content of each sample was quantified using a BCA Protein Assay Kit (Thermo Fisher Scientific). Cell lysates were then diluted, mixed with 6x Laemmli buffer (Boston BioProducts), and boiled at 95 °C for 5 min. Denatured proteins were separated on 4–12% Bis-Tris gradient gels (Thermo Fisher Scientific), and then transferred to nitrocellulose membranes (Cytiva) using the standard wet transfer method. Membranes were blocked with 5% milk and probed with the indicated antibodies. The horseradish peroxidase (HRP)-conjugated secondary antibodies and enhanced chemiluminescence (ECL) substrate (Cytiva) were applied to visualize the protein of interest. Cell fractionation was performed using a Subcellular Protein Fractionation Kit (Thermo Fisher Scientific) according to the manufacturer’s instructions. Cells underwent a stepwise lysis into cytosolic, membrane, and nuclear soluble extracts, and then were subjected to Western blotting as above. Immunoprecipitation was performed as described [29]. Briefly, cells were lysed in lysis buffer (50 mM Tris at pH 7.5, 10% glycerol, 5 mM MgCl_2_, 150 mM NaCl, 0.2% NP-40, protease (Roche) and phosphatase (Sigma-Aldrich) inhibitor) and the lysates were incubated with anti-PTEN (138G6, 1:50) antibody overnight at 4 °C. The protein G sepharose (Cytiva) was then added and incubated for another 2 h. The immunoprecipitates were washed with wash buffer (50 mM Tris at pH 7.5, 5 mM MgCl_2_, 150 mM NaCl, 0.1% NP-40, protease (Roche) and phosphatase (Sigma-Aldrich) inhibitor) three times and eluted with 2XSDS sample buffer. Densitometry quantification was performed with ImageJ. See complete unedited blots in the Supplemental Materials.

### 2.4. Immunofluorescence

Immunofluorescence was performed as described [29]. Briefly, cells were grown on coverslips (Thermo Fisher Scientific), fixed with 4% paraformaldehyde and permeabilized with ice-cold methanol. Cells were rinsed with PBS, blocked with 10% goat serum and then incubated with anti-PTEN (Sigma-Aldrich, 6H2.1, 1:100) antibody overnight, followed by incubation with GFP-conjugated secondary antibodies (Thermo Fisher Scientific, 1:500). Coverslips were mounted with ProLong Gold Antifade reagent with DAPI (Thermo Fisher Scientific). The stained slides were visualized using a confocal microscope. 

### 2.5. Targeted Polar Metabolomics via Selected Reaction Monitoring (SRM) Tandem Mass Spectrometry

Metabolomics were performed as described [30]. Briefly, one 10 cm^2^ plate of prostate cancer cells per sample were extracted with 80% methanol (−80 °C) for 20 min. Dried metabolite pellets were re-suspended in 20 μL LC/MS grade water, 5 μL were injected over a 15 min gradient using a 5500 QTRAP triple quadrupole mass spectrometer (AB/SCIEX) coupled to a Prominence UFLC HPLC system (Shimadzu, Columbia, MD, USA) via SRM of a total of 287 SRM transitions using positive/negative polarity switching corresponding to 258 unique endogenous water-soluble metabolites. The dwell time was 3 ms per SRM resulting in ∼10–14 data points acquired per detected metabolite. Samples were separated using an Amide XBridge HPLC hydrophilic interaction liquid chromatographic (HILIC) column (3.5 μm; 4.6 mm inner diameter (i.d.) × 100 mm length; Waters) at 300 μL/min. Gradients were run starting from 85% buffer B (LC/MS grade acetonitrile) to 40% B from 0 to 5 min; 40% B to 0% B from 5 to 16 min; 0% B was held from 16 to 24 min; 0% B to 85% B from 24 to 25 min; 85% B was held for 7 min to re-equilibrate the column. Buffer A was comprised of 20 mM ammonium hydroxide/20 mM ammonium acetate (pH = 9.0) in 95:5 water/acetonitrile. Peak areas from the total ion current for each metabolite SRM transition were integrated using MultiQuant version 2.1 software (AB/SCIEX) via the MQ4 peak integration algorithm using a minimum of eight data points with a 20 sec retention time window.

All metabolites undetected in at least one group of samples were excluded from the analysis. This pruning step reduced the number of metabolites to 262 and 273 in PC3 and C4-2 cells, respectively. A heatmap and principal components analysis (PCA) were carried out using R programming (http://www.r-project.org, accessed on 4 March 2021). The original values of each metabolite were standardized to have a mean of 0 and a standard deviation of 1. The PCA was performed with all metabolites used to generate the heatmap of all metabolites. Each sample was also standardized following the standardization of each metabolite. The processed table was used to compute the covariance matrix of each sample. Eigenvalues were calculated by using the covariance matrix. The eigenvector corresponding to the largest eigenvalue was PC_1. PC_2 was the eigenvector corresponding to the second-largest eigenvalue. PCA analysis was implemented using R-4.1.2 function stats::*prcomp* by setting *scale = TRUE*. Over-representation analysis of the metabolic pathways based on the human KEGG (www.genome.jp/kegg/, accessed on 9 March 2021) metabolic pathway database was carried out using MetaboAnalyst 5.0 (http://www.metaboanalyst.ca, accessed on 9 March 2021). The metabolites that significantly differed between the control and PTEN-overexpressed groups were used for the pathway analysis and are shown in Tables S3–S5 and S7–S9.

### 2.6. Statistical Analysis

For analysis of average data, an unpaired two-tailed Student’s *t* test was used for comparison of the two experimental groups. *p* values of <0.05 were considered statistically significant. Statistical tests were executed using GraphPad Prism software.

## 3. Results

### 3.1. Establishment and Validation of Prostate Cancer Cell Lines Inducibly Overexpressing PTEN Targeted to the Plasma Membrane and Nucleus

**Figure 1:**
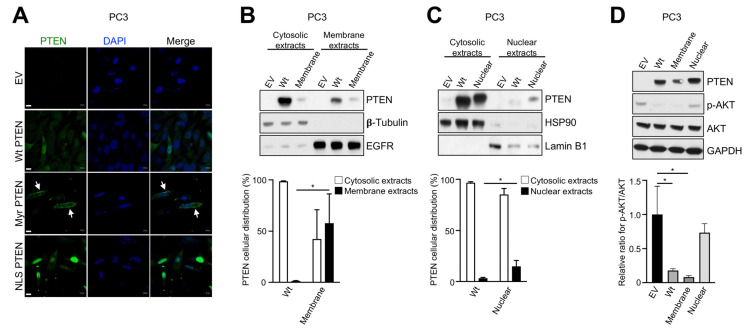
Establishment and validation of prostate cancer cell line inducibly overexpressing PTEN targeted to the plasma membrane and nucleus. (**A**) Immunofluorescent staining of PTEN in PTEN-inducible PC3 stable cell lines. Myr, myristoylated. Arrows indicate focal PTEN staining at the plasma membrane. Scale bars, 10 μm. (**B**–**D**) Immunoblot (IB) analysis of PC3 lysates from cytosolic and membrane extracts (**B**), cytosolic and nuclear extracts (**C**), or regular whole-cell extracts (**D**). In (**A**–**D**), EV, empty vector. In (**B**,**C**), quantification of the band intensity was carried out with ImageJ. After being normalized against its respective markers, the percentage of PTEN protein in cytosolic, membrane, or nuclear extracts was calculated against total PTEN protein. * *p* < 0.05. All data are mean ± SD from *n* = 3 biological replicates.

We sought to determine the impacts on cellular metabolism by PTEN in its two active sub-cellular compartments: the plasma membrane and nucleus [1]. Due to the technical challenges of compartment-specific metabolic profiling [31], our strategy was to express PTEN targeted to different subcellular compartments in *PTEN*-null prostate cancer cell lines to assess its impact on whole-cell metabolomics. To this end, using *PTEN*-null prostate cancer cell lines, PC3 and C4-2, we established PTEN inducibly-overexpressing cell lines that targeted PTEN to the plasma membrane and nucleus, respectively. The empty vector and Wt PTEN inducibly-overexpressing cell lines were also established and used as controls. Membrane PTEN contained an N-terminal myristoylation motif of c-Src, a commonly used signal targeting proteins to the plasma membrane [32]. Nuclear PTEN contained a C-terminal NLS motif of c-Myc [29]. Immunofluorescence of PC3 cells inducibly expressing PTEN showed that Wt PTEN displayed a diffused staining pattern throughout the body of the cell; membrane PTEN displayed enhanced focal membrane staining, while nuclear PTEN overlapped with DAPI to a large extent, an indication of strong nuclear localization (Figure 1A). Subcellular fractionation of cell lysates into cytosolic, membrane, and nuclear extracts indicated that, compared to PC3 cells inducibly expressing Wt PTEN, PC3 cells inducibly expressing membrane PTEN showed higher expression of PTEN in membrane extractions, while PC3 cells inducibly expressing nuclear PTEN had higher expression of PTEN in nuclear extractions (Figure 1B,C). Similar results were also obtained in C4-2 cells (Figure S1A–C). As expected, overexpression of Wt and membrane PTEN suppressed AKT phosphorylation in PC3 cells, while overexpression of nuclear PTEN had a negligible effect on the level of AKT phosphorylation (Figure 1D). Moreover, we observed that Wt and membrane PTEN had largely similar effects on suppression of AKT phosphorylation in C4-2 cells. Interestingly, overexpression of nuclear PTEN in C4-2 also strongly suppressed AKT phosphorylation, suggesting that a nuclear pool of PIP_3_ may exist in C4-2 cells that are sensitive to catalysis by nuclear PTEN, leading to suppression of AKT phosphorylation [33].

### 3.2. Targeted Metabolomics Reveals That Nuclear PTEN Induces Distinct Metabolic Changes from Wt and Membrane PTEN

**Figure 2:**
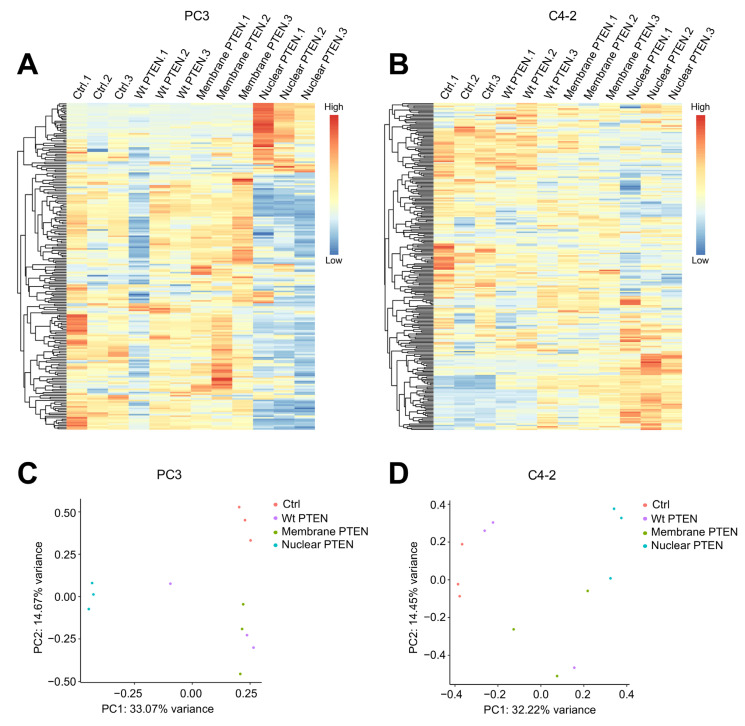
The analysis of metabolite composition in control and PTEN-overexpressed prostate cancer cells. (**A**,**B**) Unsupervised hierarchical cluster analysis by heatmap using all identified metabolites in control and PTEN-overexpressed PC3 (**A**) and C4-2 (**B**) cells. (**C**,**D**) PCA to project individual samples onto the first two principal components in control and PTEN-overexpressed PC3 (**C**) and C4-2 (**D**) cells.

To gain insight into how PTEN in different subcellular compartments influences cell metabolism, we performed a targeted polar metabolomics profiling in control and PTEN-overexpressed PC3 and C4-2 cell lines by using a liquid chromatography/tandem mass spectrometry (LC-MS/MS)-based platform [30]. This platform can detect metabolites covering all major metabolic pathways, including glycolysis, the tricarboxylic acid cycle, the pentose–phosphate pathway, and metabolism of amino acids, nucleotides, and so on (Table S1). Using this metabolomic platform, we identified 262 and 273 unique metabolites in PC3 and C4-2, respectively (Tables S2 and S6, respectively). We next performed unsupervised hierarchical clustering to analyze global differences across all the identified metabolites in control, Wt, membrane, and nuclear PTEN-overexpressed PC3 and C4-2 cells. Interestingly, the heatmap generated from the unsupervised hierarchical cluster analysis revealed that prostate cancer cells overexpressing nuclear PTEN displayed the most distinctive metabolic profiling compared to the other groups (Figure 2A,B). Likewise, PCA analysis showed that the biological triplicates for each condition clustered separately, although Wt PTEN-overexpressed cells displayed the most variation. Notably, nuclear PTEN-overexpressed prostate cancer cells formed a single cluster that was significantly separated in distance from control samples, while Wt and membrane PTEN-overexpressed cells were clustered between control and nuclear PTEN-overexpressed samples (Figure 2C,D and Figure S2A,B). These data indicate that nuclear PTEN plays a distinct role in metabolic regulation.

### 3.3. Nuclear PTEN Regulates Pyrimidine Metabolism, In Particular De Novo dTMP Biosynthesis

To assess the impacts of PTEN on specific metabolites and metabolic pathways, we analyzed significantly changed metabolites in each PTEN-overexpressed cell line compared to its control counterpart using more stringent two-tailed *t* test statistics (*p* < 0.05) (Tables S3–S5 and S7–S9). We found that, compared to control cells, 28, 33, and 105 metabolites out of 262 identified metabolites were significantly altered in Wt, membrane, and nuclear PTEN-overexpressed PC3 cells, respectively (Table S3), while 38, 65, and 114 metabolites out of 273 identified metabolites were significantly altered in Wt, membrane, and nuclear PTEN-overexpressed C4-2 cells, respectively (Table S7). Overexpression of nuclear PTEN resulted in the highest number of significantly changed metabolites among three PTEN variants, further corroborating a distinct role for nuclear PTEN in metabolic reprogramming.

**Figure 3:**
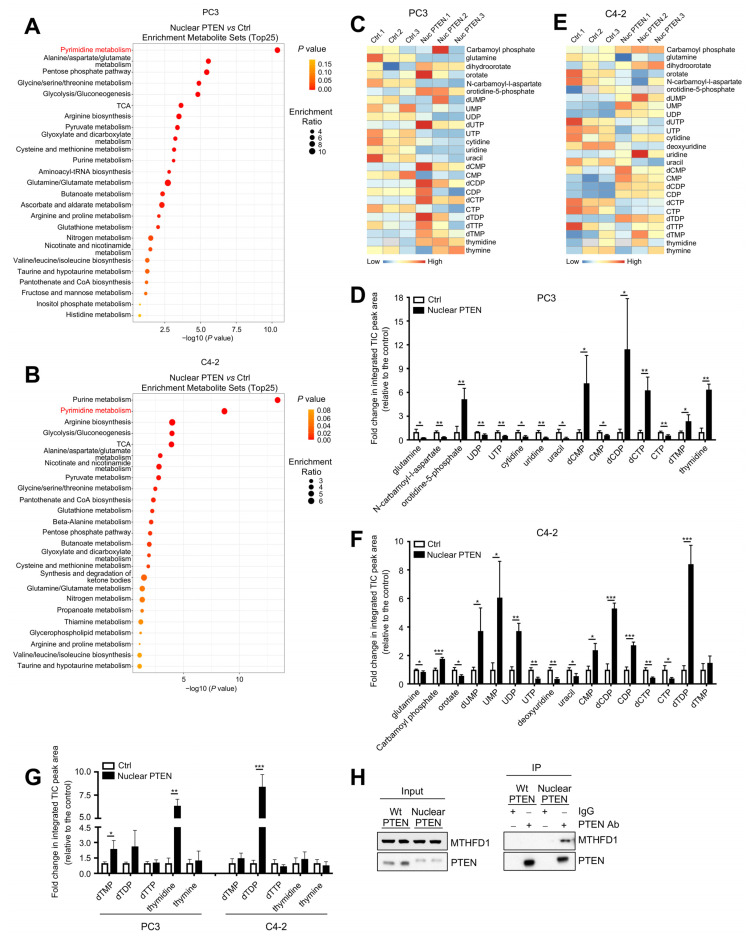
Nuclear PTEN regulates pyrimidine metabolism, specifically *de novo* dTMP biosynthesis through interaction with MTHFD1. (**A**,**B**) Over-representation analysis of significantly changed metabolites in nuclear PTEN-overexpressed PC3 (**A**) and C4-2 (**B**) cells, compared to its control counterpart. Note that pyrimidine metabolism is the most significantly enriched metabolic pathway in both nuclear PTEN-overexpressed PC3 and C4-2 cells. (**C**,**D**) Heatmap (**C**) and bar graph (**D**) showing the relative abundance of various significantly altered metabolites in the pyrimidine metabolic pathway in nuclear PTEN-overexpressed PC3 cells. (**E**,**F**) Heatmap (**E**) and bar graph (**F**) showing the relative abundance of various significantly altered metabolites in the pyrimidine metabolic pathway in nuclear PTEN-overexpressed C4-2 cells. (**G**) Bar graph showing the relative abundance of dTMP and its immediate downstream metabolites in both nuclear PTEN-overexpressed PC3 and C4-2 cells. (**H**) Co-immunoprecipitation of PTEN and MTHFD1 in Wt and nuclear PTEN-overexpressed PC3 cells using control IgG and PTEN antibody. Input is 10% of total cell extracts used for immunoprecipitation. * *p* < 0.05, ** *p* < 0.01, *** *p* < 0.001. All data are mean ± SD from *n* = 3 biological replicates.

We next performed over-representation analysis of significantly altered metabolites using MetaboAnalyst 5.0. Consistent with the results obtained in the hierarchical clustering and PCA analyses, Wt and membrane PTEN-overexpressed PC3 and C4-2 cells shared several commonly enriched metabolic pathways (6 out of top 25 regulated metabolic pathways, Figure S2C–F). Among them, glycolysis and purine metabolism, two metabolic pathways known to be regulated by *PTEN* loss and PI3K/AKT activation [20,21,34], were among the top 10 altered metabolic pathways in all Wt and membrane PTEN-overexpressed cell lines (Figure S2C–F). These data indicate the validity of our assays and suggest that membrane PTEN regulates cellular metabolism through its canonical functions in a manner similar to Wt PTEN. Strikingly, nuclear PTEN-overexpressed PC3 and C4-2 cells shared 80% of top regulated metabolic pathways (20 out of top 25 regulated metabolic pathways, Figure 3A,B). Among them, pyrimidine metabolism is the highest enriched pathway in nuclear PTEN-overexpressed PC3 cells and the second-highest enriched pathway in nuclear PTEN-overexpressed C4-2 cells. Furthermore, among 39 metabolites in the pyrimidine metabolite set used by MetaboAnalyst 5.0, 15 metabolites were significantly altered by overexpression of nuclear PTEN in both PC3 (Figure 3C,D) and C4-2 (Figure 3E,F) cells, compared to the control cells. Notably, among significantly altered metabolites, dTMP and its immediate downstream metabolites, including dTDP and thymidine, were all upregulated in nuclear PTEN-overexpressed PC3 and C4-2 cells, compared to control cells (Figure 3G), suggesting that nuclear PTEN promotes *de novo* dTMP biosynthesis. These data indicate that nuclear PTEN drives significant metabolic reprogramming of pyrimidine metabolism, including *de novo* dTMP biosynthesis.

The conversion of dUMP to dTMP is the sole *de novo* pathway for production of dTMP and is catalyzed by TYMS in the nucleus with the assistance of the enzymes MTHFD1 or serine hydroxymethyltransferase, which generates 5,10-methylenetetrahydrofolate as the one-carbon donor for TYMS. MTHFD1 contributes to >70% of 5,10-methylenetetrahydrofolate as the one-carbon donor and is essential for production of dTMP [35]. We previously compiled a list of PTEN-associated proteins through Mass-Spec, and identified MTHFD1 as a candidate PTEN-interacting protein [2]. Through immunoprecipitation, we confirmed that nuclear PTEN, but not Wt PTEN, could co-immunoprecipitate MTHFD1 in PTEN-overexpressed PC3 cell lines (Figure 3H), indicating that nuclear PTEN may regulate dTMP biosynthesis through interaction with MTHFD1. 

## 4. Discussion

We mapped the different impacts on global metabolism by PTEN in the cytoplasm and nucleus of human prostate cancer cells by using a targeted mass spectrometry-based polar metabolomics profiling platform. This platform was designed to include as many polar metabolites (Table S1) as possible to cover all the major metabolic pathways potentially involved in cancer cell metabolism. We robustly quantified the relative levels of 262 and 273 unique water-soluble metabolites in control and PTEN-overexpressed PC3 and C4-2 cells, respectively. In addition to observing the enrichment of known metabolic pathways regulated by *PTEN* loss and PI3K/AKT activation in Wt and membrane PTEN-overexpressed samples, our data have revealed a novel role for nuclear PTEN in the regulation of pyrimidine metabolism, and specifically the enhancement of dTMP biosynthesis. Impaired *de novo* dTMP synthesis is known to increase rates of uracil misincorporation into DNA, leading to genomic instability [26,27,28]. As PTEN is a guardian of genome integrity [1], our study suggests a potential new mechanism underlying PTEN-mediated genomic stability. Additionally, increased *TYMS* gene expression and *de novo* dTMP synthesis are associated with poor response to antifolate treatment [25,36]. As aberrant PTEN subcellular localization, including loss of nuclear PTEN, is often observed in human cancer [6,7,8], further studies are needed to determine whether PTEN nuclear-excluded mutant cancer cells are susceptible to cell killing by antifolate drugs in vitro and in vivo.

Because the metabolic network relies on many common intermediates for macromolecule biosynthesis, metabolic pathways must be compartmentalized to avoid futile cycles and to direct metabolic flow towards the correct output. Metabolic compartmentalization can be achieved through the formation of local enzyme and protein complexes. Our study indicates that PTEN may form a nuclear complex with MTHFD1 to assist dTMP synthesis in the nucleus. Further mechanistic studies are needed to evaluate how PTEN may impact the enzyme activity of MTHFD1, or other key enzymes involved in dTMP synthesis. Given that many metabolic enzymes are reported to play non-metabolic functions [37], it would also be interesting to explore how MTHFD1 may affect the functionality of PTEN. 

Our study has some limitations. The metabolic pathway is a complex network. PTEN may impact metabolic pathways at multiple nodes with opposite effects. More mechanistic work is needed to define the specific metabolic conversion and metabolic enzymes that are impacted by PTEN in a metabolic pathway. Together, our results reveal that nuclear PTEN induces metabolic changes, including dTMP biosynthesis, that are distinct from Wt and membrane PTEN, and provide an important data resource for metabolites that may potentially be regulated by PTEN in human prostate cancer cells.

## Data Availability

All data supporting the findings of this study are available within the article and its Supplementary Materials and are available from the authors upon reasonable request.

## Supplementary Materials

The following supporting information can be downloaded at: https://www.mdpi.com/article/10.3390/metabo13080939/s1, Figure S1: Establishment and validation of prostate cancer cell line inducibly overexpressing PTEN targeted to the plasma membrane and nucleus; Figure S2: Wt and membrane PTEN shared several commonly enriched metabolic pathways; Table S1: Name, formula, and PubChem identifier of detectable polar metabolites; Tables S2–S5: Results of polar metabolomics in Ctrl and PTEN-overexpressed PC3 cells; Tables S6–S9: Results of polar metabolomics in Ctrl and PTEN-overexpressed C4-2 cells.
