# Supplementary material for: Nuclear PTEN Regulates Thymidylate Biosynthesis in Human Prostate Cancer Cell Lines
Source: Metabolites. 2023 Aug 11;13(8):939. doi: 10.3390/metabo13080939 (PMC10456368; doi:10.3390/metabo13080939)
Supplement: Supplementary file 1 [file metabolites-13-00939-s001.zip › Supplementary data.pdf]

Supplementary Data

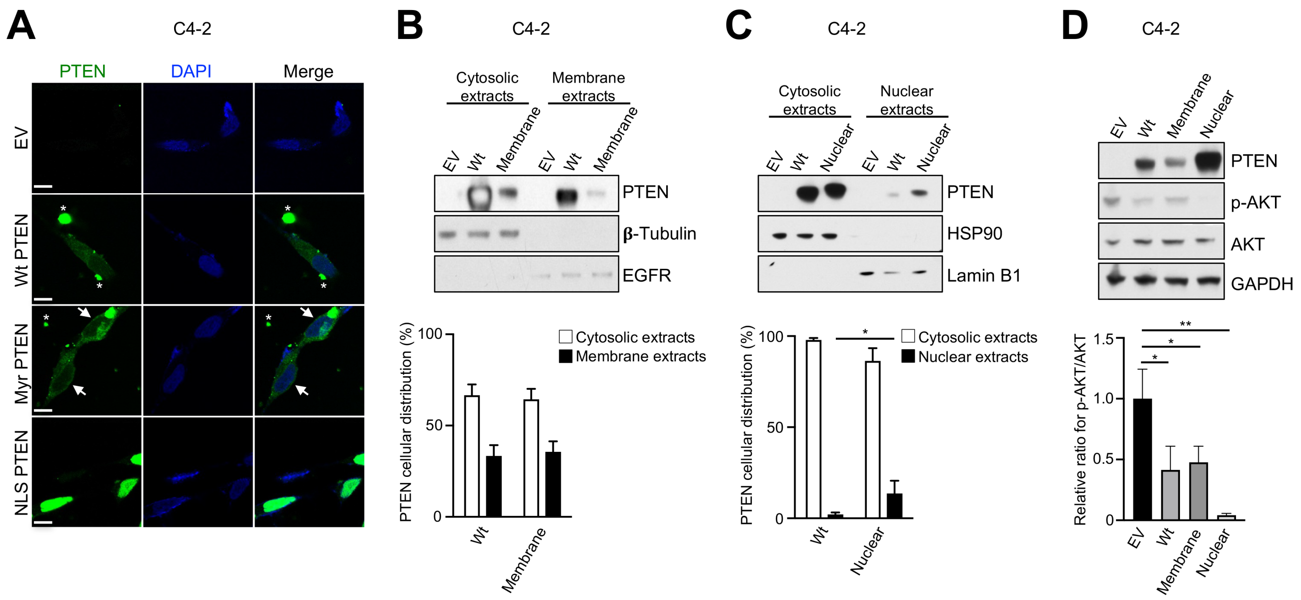

Supplementary Figure 1. Establishment and validation of prostate cancer cell line inducibly overexpressing PTEN targeted to the plasma membrane and nucleus. **(A)** Immunofluorescent staining of PTEN in PTEN inducible C4-2 stable cell lines. Myr, myristoylated. Arrows indicate focal PTEN staining at the plasma membrane. Stars indicate non-specific fluorescent dye staining. Scale bars, 20 µm. (**B–D**) IB analysis of C4-2 lysates from cytosolic and membrane extracts (**B**), cytosolic and nuclear extracts (**C**), or regular whole-cell extracts (**D**). In **A–D**, EV, empty vector. In **B** and **C**, quantification of the band intensity was carried out with ImageJ. After normalized against its respective markers, the percentage of PTEN protein in cytosolic, membrane, or nuclear extracts was calculated against total PTEN protein. *$P < 0.05$, **$P < 0.01$. All data are mean ± SD from $n = 3$ biological replicates.

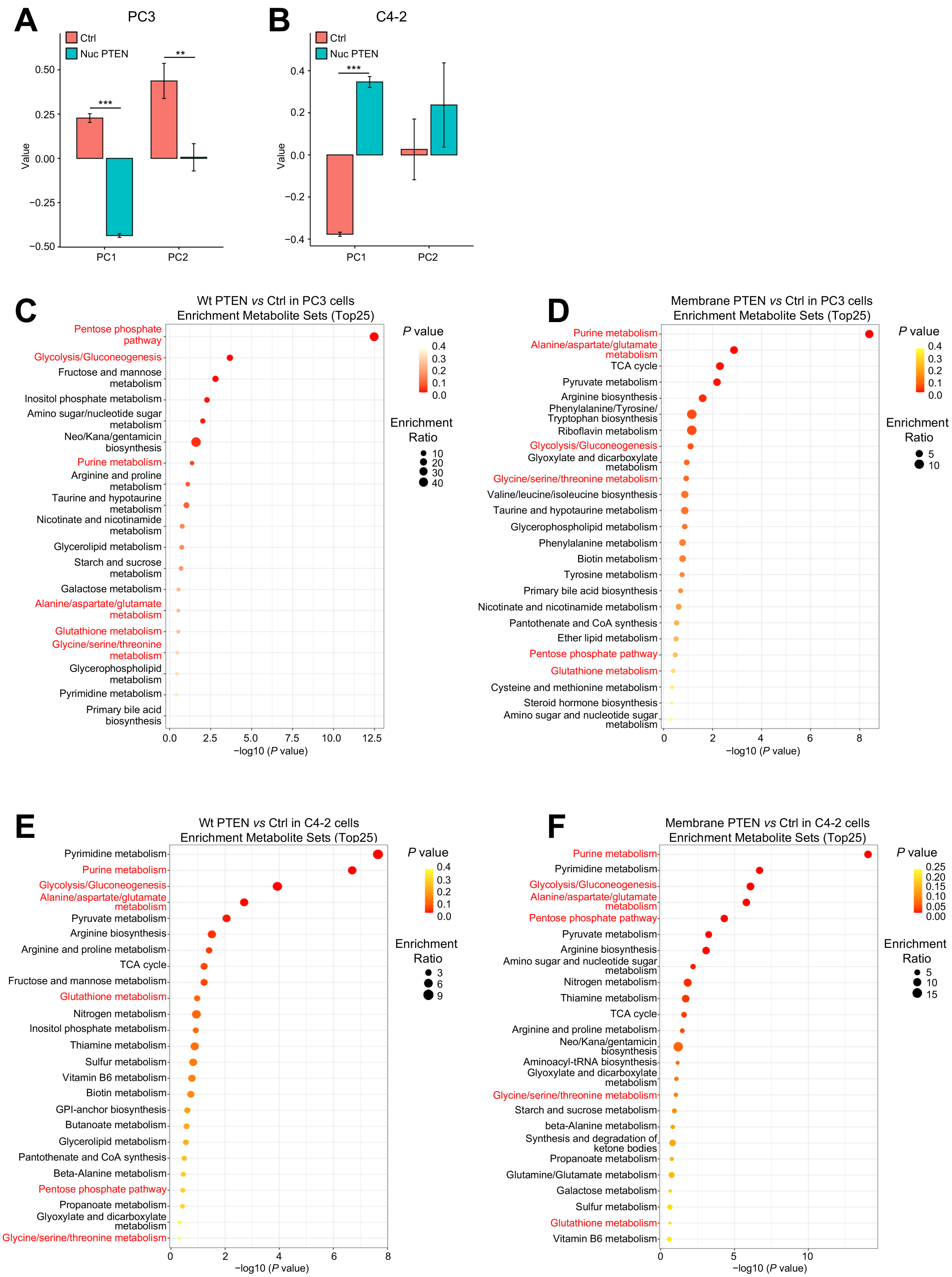

A
PC3
Ctrl
Nuc PTEN
***
**
Value
0.50
0.25
0.00
−0.25
−0.50
PC1
PC2
B
C4-2
Ctrl
Nuc PTEN
***
Value
0.4
0.2
0.0
−0.2
−0.4
PC1
PC2
C
Wt PTEN vs Ctrl in PC3 cells
Enrichment Metabolite Sets (Top25)
Pentose phosphate pathway
Glycolysis/Gluconeogenesis
Fructose and mannose metabolism
Inositol phosphate metabolism
Amino sugar/nucleotide sugar metabolism
Neo/Kana/gentamicin biosynthesis
Purine metabolism
Arginine and proline metabolism
Taurine and hypotaurine metabolism
Nicotinate and nicotinamide metabolism
Glycerolipid metabolism
Starch and sucrose metabolism
Galactose metabolism
Alanine/aspartate/glutamate metabolism
Glutathione metabolism
Glycine/serine/threonine metabolism
Glycerophospholipid metabolism
Pyrimidine metabolism
Primary bile acid biosynthesis
0.0
2.5
5.0
7.5
10.0
12.5
−log10 (P value)
P value
0.4
0.3
0.2
0.1
0.0
Enrichment Ratio
10
20
30
40
D
Membrane PTEN vs Ctrl in PC3 cells
Enrichment Metabolite Sets (Top25)
Purine metabolism
Alanine/aspartate/glutamate metabolism
TCA cycle
Pyruvate metabolism
Arginine biosynthesis
Phenylalanine/Tyrosine/ Tryptophan biosynthesis
Riboflavin metabolism
Glycolysis/Gluconeogenesis
Glyoxylate and dicarboxylate metabolism
Glycine/serine/threonine metabolism
Valine/leucine/isoleucine biosynthesis
Taurine and hypotaurine metabolism
Glycerophospholipid metabolism
Phenylalanine metabolism
Biotin metabolism
Tyrosine metabolism
Primary bile acid biosynthesis
Nicotinate and nicotinamide metabolism
Pantothenate and CoA synthesis
Ether lipid metabolism
Pentose phosphate pathway
Glutathione metabolism
Cysteine and methionine metabolism
Steroid hormone biosynthesis
Amino sugar and nucleotide sugar metabolism
0
2
4
6
8
−log10 (P value)
P value
0.4
0.3
0.2
0.1
0.0
Enrichment Ratio
5
10
E
Wt PTEN vs Ctrl in C4-2 cells
Enrichment Metabolite Sets (Top25)
Pyrimidine metabolism
Purine metabolism
Glycolysis/Gluconeogenesis
Alanine/aspartate/glutamate metabolism
Pyruvate metabolism
Arginine biosynthesis
Arginine and proline metabolism
TCA cycle
Fructose and mannose metabolism
Glutathione metabolism
Nitrogen metabolism
Inositol phosphate metabolism
Thiamine metabolism
Sulfur metabolism
Vitamin B6 metabolism
Biotin metabolism
GPI-anchor biosynthesis
Butanoate metabolism
Glycerolipid metabolism
Pantothenate and CoA synthesis
Beta-Alanine metabolism
Pentose phosphate pathway
Propanoate metabolism
Glyoxylate and dicarboxylate metabolism
Glycine/serine/threonine metabolism
0
2
4
6
8
−log10 (P value)
P value
0.4
0.3
0.2
0.1
0.0
Enrichment Ratio
3
6
9
F
Membrane PTEN vs Ctrl in C4-2 cells
Enrichment Metabolite Sets (Top25)
Purine metabolism
Pyrimidine metabolism
Glycolysis/Gluconeogenesis
Alanine/aspartate/glutamate metabolism
Pentose phosphate pathway
Pyruvate metabolism
Arginine biosynthesis
Amino sugar and nucleotide sugar metabolism
Nitrogen metabolism
Thiamine metabolism
TCA cycle
Arginine and proline metabolism
Neo/Kana/gentamicin biosynthesis
Aminoacyl-tRNA biosynthesis
Glyoxylate and dicarboxylate metabolism
Glycine/serine/threonine metabolism
Starch and sucrose metabolism
beta-Alanine metabolism
Synthesis and degradation of ketone bodies
Propanoate metabolism
Glutamine/Glutamate metabolism
Galactose metabolism
Sulfur metabolism
Glutathione metabolism
Vitamin B6 metabolism
0
5
10
−log10 (P value)
P value
0.25
0.20
0.15
0.10
0.05
0.00
Enrichment Ratio
5
10
15

Supplementary Figure 2. Wt and membrane PTEN shared several commonly enriched metabolic pathways. **(A, B)** The statistical analysis of the first and second components between control and nuclear PTEN-overexpressed PC3 (**A**) and C4-2 cells (**B**). (**C, D**) Over-representation analysis of significantly changed metabolites in Wt PTEN (**C**) and membrane PTEN (**D**) overexpressed PC3 cells, compared to its control counterpart. (**E, F**) Over-representation analysis of significantly changed metabolites in Wt PTEN (**E**) and membrane PTEN (**F**) overexpressed C4-2 cells, compared to its control counterpart. Six commonly enriched metabolic pathways, including glycolysis and purine metabolism, in all Wt and membrane PTEN-overexpressed cell lines are highlighted in Red. $^{**}P < 0.01$, $^{***}P < 0.001$. All data are mean ± SD from $n = 3$ biological replicates.
